# Ovarian Ectopic Pregnancy, an Unusual Diagnosis—Two Cases With Two Distinct Treatment Approaches

**DOI:** 10.1002/ccr3.71380

**Published:** 2025-11-19

**Authors:** Andreia Mota de Sousa, António de Pinho, Rosete Nogueira, Anabela Melo, Isabel Meireles, Elisa Soares, Olímpia do Carmo, Diana Melo e Castro

**Affiliations:** ^1^ Serviço de Ginecologia e Obstetrícia Unidade Local de Saúde do Tâmega e Sousa Penafiel Portugal; ^2^ Departamento de Ginecologia‐Obstetrícia e Pediatria da Faculdade de Medicina da Universidade do Porto Porto Portugal; ^3^ Escola de Medicina Universidade do Minho Braga Portugal; ^4^ Instituto de Investigação em Ciências da Vida e Saúde, ICVS/3B's‐PT Braga Portugal; ^5^ Laboratório de Patologia Placentar e Embriofetal, CGC Unilabs Porto Portugal

**Keywords:** ectopic pregnancy, gynecological emergency, minimal invasive surgery, ovarian pregnancy

## Abstract

Ovarian pregnancy is a rare event, nevertheless, it poses a potential life‐threatening gynecological emergency. The clinical history and objective exam are essential, however for the majority of cases, the diagnosis is performed intraoperatively, with the treatment approach only being determined during surgery. In this article, we describe two cases of ovarian pregnancies, where distinct surgical approaches were performed.

**Abbreviations**
EP: Ectopic pregnancy
bHCG: beta‐Human chorionic gonadotropin
H&E: Hematoxylin and eosin stain
OP: Ovarian pregnancy

**Summary**

- Despite its low incidence, ovarian pregnancy represents a potential life‐threatening gynecological emergency, in women of reproductive age.
- Early diagnosis and prompt treatment are imperative, and minimally invasive surgery, namely the fertility‐sparing approach, should be prioritized whenever feasible.

## 1 Introduction

Ectopic pregnancy (EP) represents around 2% of all pregnancies, but true incidence is difficult to estimate since surveillance data is lacking [1]. EP can present as a variety of symptoms such as amenorrhea or abdominal/pelvic pain with or without vaginal bleeding. More unspecific symptoms can also be present, including gastrointestinal or urinary symptoms, dizziness, fainting or syncope, rectal pressure/pain on defecation, among others [2]. Although rare, EP‐associated mortality accounts for 2.7% of all pregnancy‐related deaths, which can be attributed to challenges with diagnosis and treatment delays [3].

The most common EP location is, in 96% of the cases, the fallopian tube. Nevertheless, other locations can occur, namely the abdomen, cervix, cesarean scar and ovary. Ovarian pregnancy (OP) accounts for 1%–3% of all ectopic pregnancies. There are some risk factors described as being associated with OP such as pelvic inflammatory disease history, assisted reproductive techniques, the use of ovarian stimulation protocols and the use of intrauterine devices [4, 5, 6, 7]. Due to the fragility and hypervascularity of the ovarian tissue, there is an increased risk of tissue rupture, which can result in significant internal bleeding, hence consisting of a potentially life‐threatening condition [7, 8].

OP represents a diagnostic challenge, since the imagiological difference between OP and a corpus luteum cyst is difficult, unless a gestational sac with an embryo is visualized surrounded by ovarian tissue. In most cases, OP is diagnosed intraoperatively, although it requires histopathologic confirmation. *Comstock* and colleagues conducted a case series in which they evaluated the ultrasound characteristics of confirmed ovarian ectopic pregnancies. It usually presents as a wide echogenic ring with a central echolucent area as compared to a thin tubal ring observed for tubal pregnancies or corpus luteum cyst [9]. Diagnostic criteria were originally described by Spiegelburg and include the following: (1) the gestational sac must be located in the normal position of the ovary; (2) the fallopian tube on the involved site must be intact; (3) the gestational sac should be connected to the uterus by the utero‐ovarian ligament; and (4) ovarian tissue needs to be identified histologically in the wall of the gestational sac [10].

The EP location does not represent a contraindication for conservative management (expectant management or methotrexate treatment), and it has been described, despite not being a common approach. Nevertheless, due to the high risk of rupture, surgical management has become the standard of care, namely, laparoscopic approach in hemodynamic stable patients.

In this article we describe two cases of ovarian pregnancies, where two distinct surgical approaches were used.

## 2 Case Presentation

### 2.1 Case 1

#### 2.1.1 Case History/Examination

**FIGURE 1:**
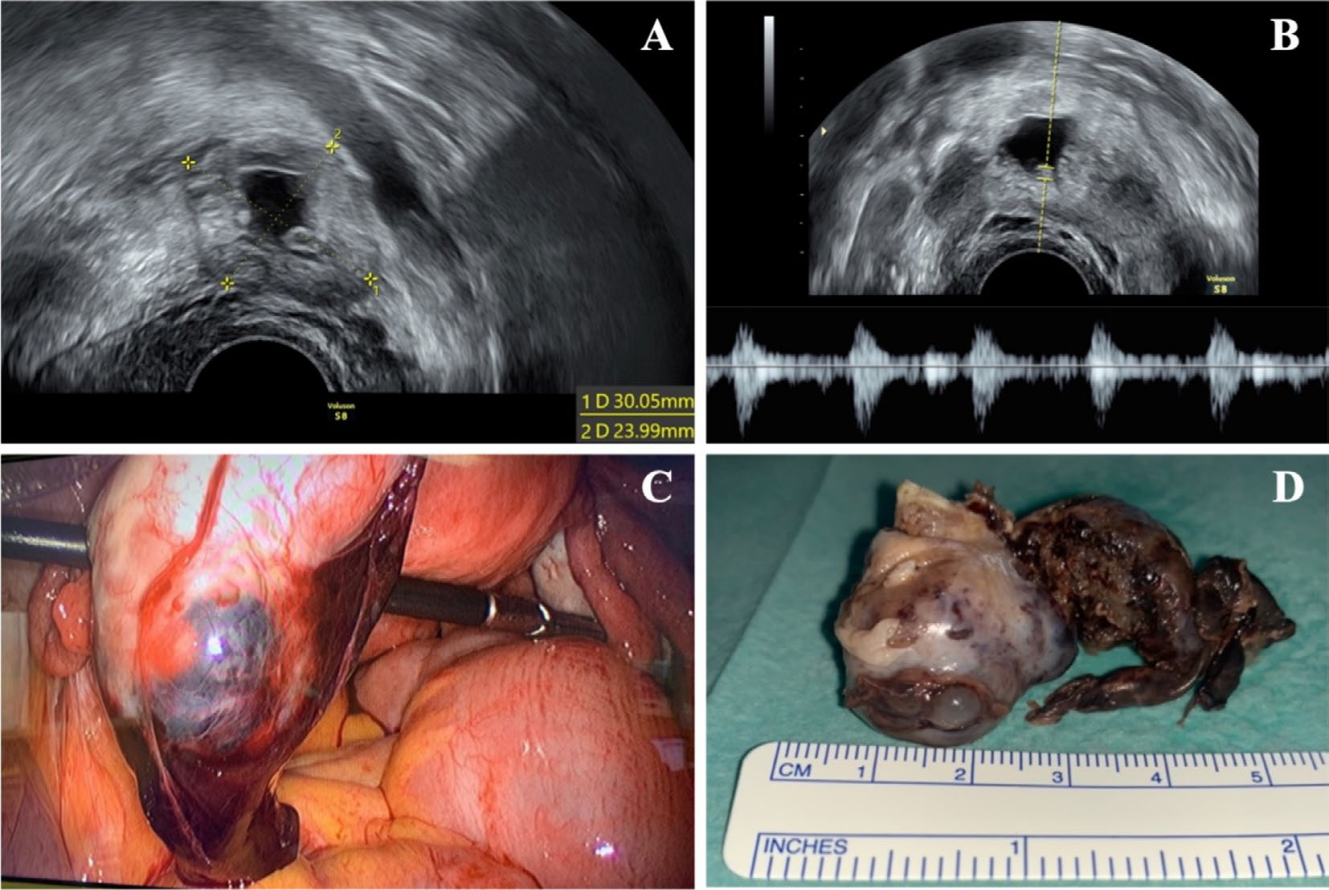
(A) Transvaginal ultrasonography with a hypoechogenic image with an echolucent region suggestive of a gestational sac; (B) Transvaginal ultrasonography image showing embryonic cardiac activity; (C) Intraoperative image of left ovary with purple‐and‐blue mass, suspected to be a gestational sac; (D) Left oophorectomy specimen.

Woman, 39‐year‐old, gravida 5 para 2 (2‐0‐2‐2), with an unremarkable medical history, presented to the emergency department after a positive pregnancy test and 6 weeks of amenorrhea. She had no complaints, and both vital signs and physical examination were unremarkable. Transvaginal ultrasound showed an empty uterine cavity and a left paraovarian image with 30 mm × 24 mm, that corresponded to a gestational sac and embryo with cardiac activity (Figures 1A,B) and no signs of intra‐abdominal hemorrhage. Serum beta‐human chorionic gonadotropin (bHCG) was 17723 mUI/mL.

#### 2.1.2 Management and Clinical Course

Left EP was presumed and laparoscopic surgery was performed. Intraoperatively, an EP was identified in the left ovary (Figure 1C,D), with an intact fallopian tube. A unilateral left oophorectomy was performed, and the specimen was sent for histopathological study.

**FIGURE 2:**
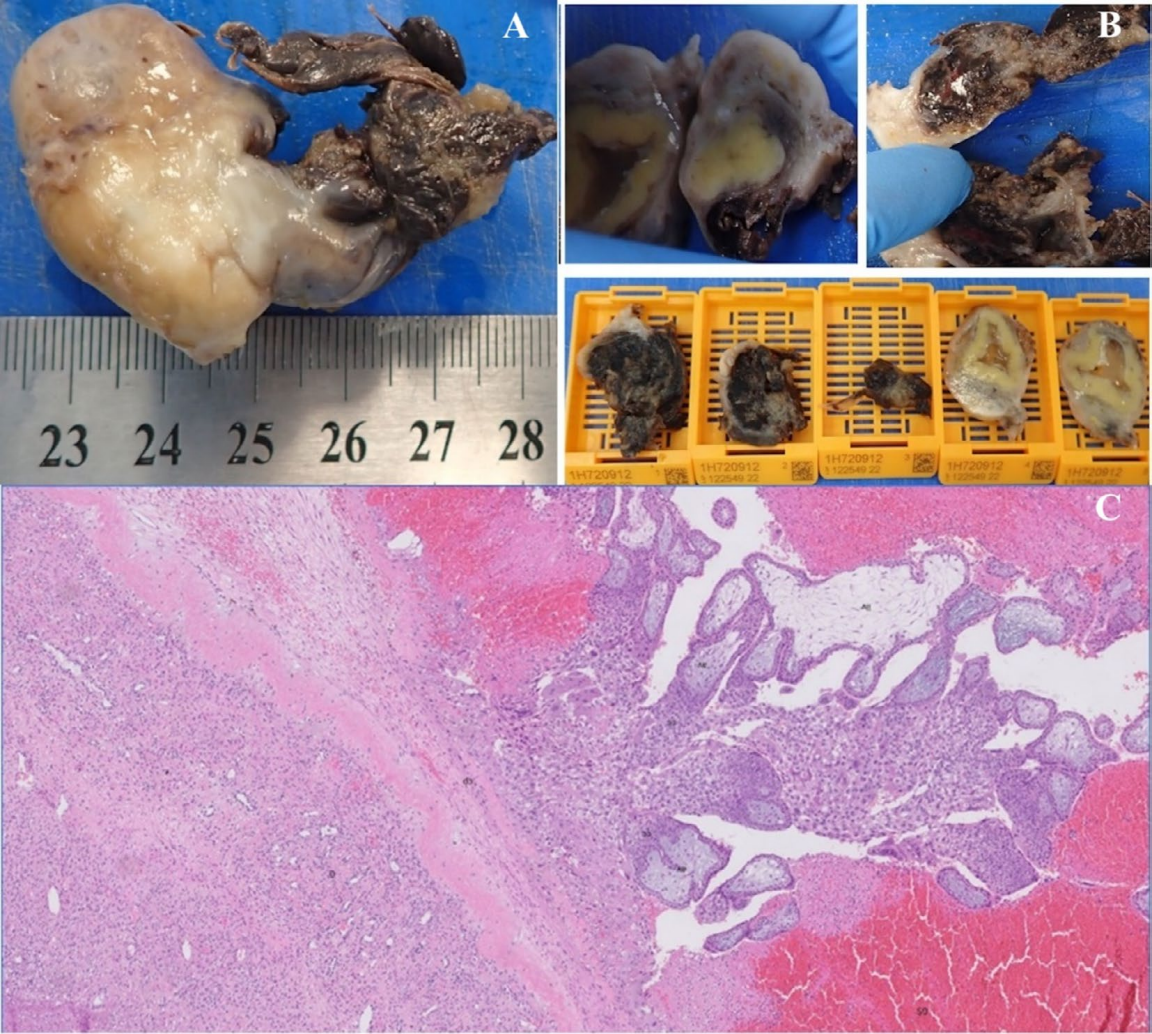
Macroscopic features (A, B): (A) Oophorectomy surgical specimen; (B) Corpus luteum, ruptured gestational sac and blocks with representative fragments for histology. Digital microscopy image (C): Ovary parenchyma with corpus luteum (left corner) contiguous with trophoblastic shell and gestational sac with anchoring villi and hyperplastic extravillous trophoblast cells (H&E).

The pathological study identified an oophorectomy specimen measuring 5.5 × 3 × 2 cm, with a corpus luteum (2 × 1.5 cm) and a gestational sac (1.5 × 1 cm) (Figure 2A,B). Microscopically, an ovary with a corpus luteum adherent to a ruptured gestational sac with a hemorrhagic trophoblastic shell and immature villi was observed (Figure 2C).

There were no complications during the postoperative period and the patient was discharged the day after surgery with a serum bHCG of 3406 mUI/mL. One month after surgery, the patient did not present any complaints, and serum bHCG was negative.

### 2.2 Case 2

#### 2.2.1 Case History/Examination

Woman, 25‐year‐old, gravida 3 para 1 (1‐0‐1‐1), was admitted to the emergency department with nausea and acute pain in the right and lower abdomen, with 1h of evolution. The patient was not using any contraceptive method since she was trying to get pregnant. She had a surgical history of one cesarean section delivery but otherwise no other relevant medical history.

The patient had normal vital parameters and low inferior abdomen tenderness. On physical examination, vaginal inspection was unremarkable, and bimanual examination was difficult due to pain. Serum bHCG level was 7100 mUI/mL. Transvaginal sonography showed an empty uterine cavity and a right heterogeneous paraovarian mass suspected to be an EP, with moderate intraperitoneal bleeding.

#### 2.2.2 Management and Clinical Course

**FIGURE 3:**
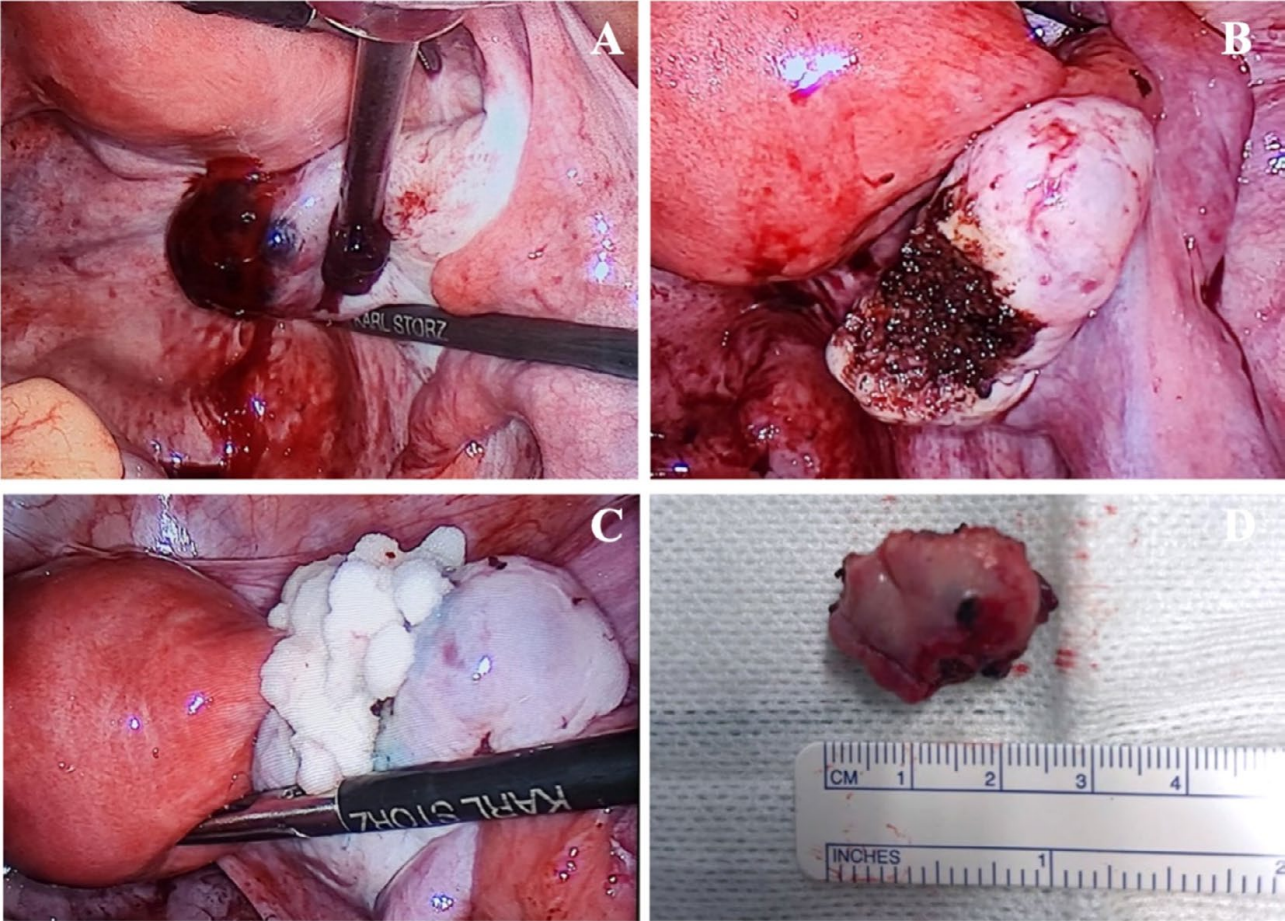
Wedge resection of suspected gestational sac. (A) Intraoperative image of purple‐and‐blue mass suspected to be an ovarian pregnancy; (B) Remaining ovary after resection; (C) Hemostatic foam over the remaining ovary; (D) Cut section of the ovary.

A right EP was suspected, and laparoscopic surgery was performed. Operative findings revealed a normal‐sized uterus and left adnexa. A purple‐and‐blue mass on the surface of the right ovary with active bleeding was observed (Figure 3A). Wedge resection of the suspected OP was performed, and a hemostatic foam was placed over the remaining ovarian tissue (Figure 3B,C). The specimen (Figure 3D) was sent for histopathological study.

**FIGURE 4:**
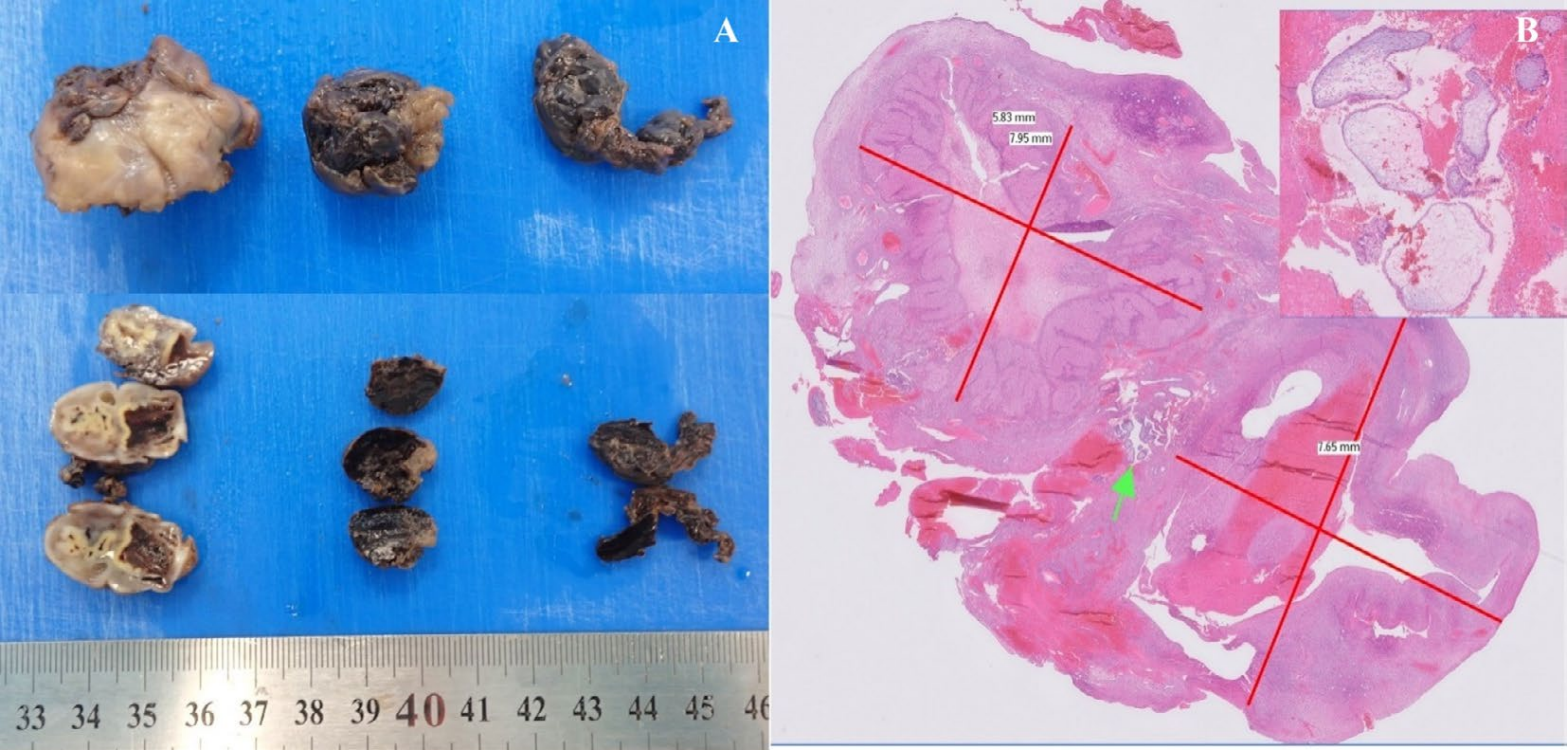
(A) Macroscopic features of the ovarian segment, clots and gestational sac within the ovarian parenchyma. (B) Digital microscopy image of the oophorectomy specimen with 2 luteomas (red lines) sequestering the gestational sac (green arrow) with hydropic chorionic villi (right corner) (H&E).

The pathological study identified an ovarian segment (2.5 × 2 × 1.5 cm), with a yellow smooth and ruptured hemorrhagic surface and hematic clots (Figure 4A). Microscopically, the clinical diagnosis was confirmed with the identification of ovarian parenchyma with luteomas (1 × 0.8 cm and 0.8 × 0.5 cm), contiguous to the gestational sac (Figure 4B).

The postoperative period was uneventful, with the patient being discharged 1 day after surgery. In a follow‐up evaluation, she had no complaints and serum bHCG was negative. Recently, the patient achieved a successful and uncomplicated pregnancy, with a live term birth 29 months after the surgery.

## 3 Discussion

OP is rare and its incidence is underestimated, since the diagnosis is challenging. Some ectopic pregnancies reported as tubal pregnancies and treated conservatively, with expectant or medical treatment, might indeed have been early OP. OP usually presents with abdominal pain, with or without vaginal bleeding. It often ends early within the first trimester, since it frequently results in premature rupture of the gestational sac with hemoperitoneum and hemorrhagic shock, due to increased ovarian tissue vascularity, hence representing a life‐threatening gynecological emergency [11]. The pathophysiology of OP is not well understood but it has been hypothesized that it could result from an ovulatory dysfunction, when the oocyte is not released and is fertilized within the follicle, or through reverse migration of the embryo after oocyte fertilization [12].

Since OP is a rare and potentially life‐threatening diagnosis, the report of cases like those herein presented, becomes crucial to gain a better understanding of this clinical entity.

The clinical presentation associated with a paraovarian ultrasound image is common, although differential diagnosis from the more common ectopic tubal location is difficult. The similar sonographic appearance with ipsilateral corpus luteum is not straightforward, making a diagnosis of OP preoperatively challenging. In order to increase the preoperative diagnostic accuracy of OP, which is currently estimated to be achieved in only around 11% of the cases, it is paramount to further develop and optimize the available diagnostic methodologies [13].

In the first case report, no risk factors were identified. The rarity of this case is emphasized by the absence of symptoms despite the presence of a gestational sac with embryonic cardiac activity, as OP is typically found to be ruptured. In the presence of a hemodynamically stable patient, like in this case, a laparoscopic surgical approach should be considered, depending on the surgeon's expertise and the available facilities.

When OP is suspected intraoperatively, oophorectomy, wedge resection of the ovary tissue with ectopic gestation or enucleation could be performed. As described in the second case report, even in the presence of hemoperitoneum, a conservative surgical approach could be an option, allowing for the maintenance of the remaining ovarian tissue, which could have a positive impact on reproductive future. Trophoblastic tissue may persist after wedge resection; therefore, follow‐up monitoring of bHCG levels should be included as part of the routine standard of care. In the reported case, no further treatment was necessary, demonstrating that a fertility‐sparing laparoscopic approach, even when OP is suspected to be ruptured, should be considered.

## 4 Conclusion

OP remains a diagnostic challenge but should always be suspected in females of reproductive age presenting with acute abdominal/pelvic pain with or without vaginal bleeding. Despite the low incidence of this pathology, the risks associated with it and its complications, including mortality, make early diagnosis and prompt treatment imperative.

## Author Contributions

**Andreia Mota de Sousa:** conceptualization, methodology, writing – original draft. **António de Pinho:** methodology, writing – review and editing. **Rosete Nogueira:** resources, writing – review and editing. **Anabela Melo:** resources, writing – review and editing. **Isabel Meireles:** methodology, writing – review and editing. **Elisa Soares:** resources, writing – review and editing. **Olímpia do Carmo:** conceptualization, supervision, writing – review and editing. **Diana Melo e Castro:** conceptualization, supervision, writing – review and editing.

## Consent

Written informed consent statements were obtained for publication.

## Conflicts of Interest

The authors declare no conflicts of interest.

## Data Availability

The authors have nothing to report.
